# ChemInformatics Model Explorer (CIME): exploratory analysis of chemical model explanations

**DOI:** 10.1186/s13321-022-00600-z

**Published:** 2022-04-04

**Authors:** Christina Humer, Henry Heberle, Floriane Montanari, Thomas Wolf, Florian Huber, Ryan Henderson, Julian Heinrich, Marc Streit

**Affiliations:** 1 Division Crop Science, Bayer AG, 40789 Monheim am Rhein, DE Germany; 2 Johannes Kepler University Linz, Linz, Austria; 3 Digital Technologies, Bayer AG, 13353 Berlin, DE Germany; 4 Division Crop Science, Bayer AG, 65926 Frankfurt, DE Germany

**Keywords:** Virtual screening, Explainable AI, Artificial intelligence, In silico, Interpretable, Explanations

## Abstract

The introduction of machine learning to small molecule research– an inherently multidisciplinary field in which chemists and data scientists combine their expertise and collaborate - has been vital to making screening processes more efficient. In recent years, numerous models that predict pharmacokinetic properties or bioactivity have been published, and these are used on a daily basis by chemists to make decisions and prioritize ideas. The emerging field of explainable artificial intelligence is opening up new possibilities for understanding the reasoning that underlies a model. In small molecule research, this means relating contributions of substructures of compounds to their predicted properties, which in turn also allows the areas of the compounds that have the greatest influence on the outcome to be identified. However, there is no interactive visualization tool that facilitates such interdisciplinary collaborations towards interpretability of machine learning models for small molecules. To fill this gap, we present CIME (ChemInformatics Model Explorer), an interactive web-based system that allows users to inspect chemical data sets, visualize model explanations, compare interpretability techniques, and explore subgroups of compounds. The tool is model-agnostic and can be run on a server or a workstation.

**Supplementary Information:**

The online version contains supplementary material available at 10.1186/s13321-022-00600-z.

## Introduction

In small molecule and drug discovery research, machine learning (ML) and exploratory data analysis techniques are crucial to making screening processes more efficient and performing quantitative structure-activity relationship (QSAR) studies. Scientists investigate sets of thousands of chemical compounds and analyze their properties, similarities, and other information using cheminformatics tools. *In silico* experiments are already part of life science research in general and have proved their value in drug discovery and design [1–3].

Predictive models enable prioritization of compounds with otherwise unknown properties and facilitate cost-effective discovery of promising candidate compounds. Further, data scientists can use explainable artificial intelligence (XAI) methods to gain insights into the reasoning underlying the models and identify chemical regions of interest. XAI techniques aim to unveil information hidden in ML models that are not readily interpretable. Making this information understandable to humans requires visualization techniques [4].

**Fig. 1:**
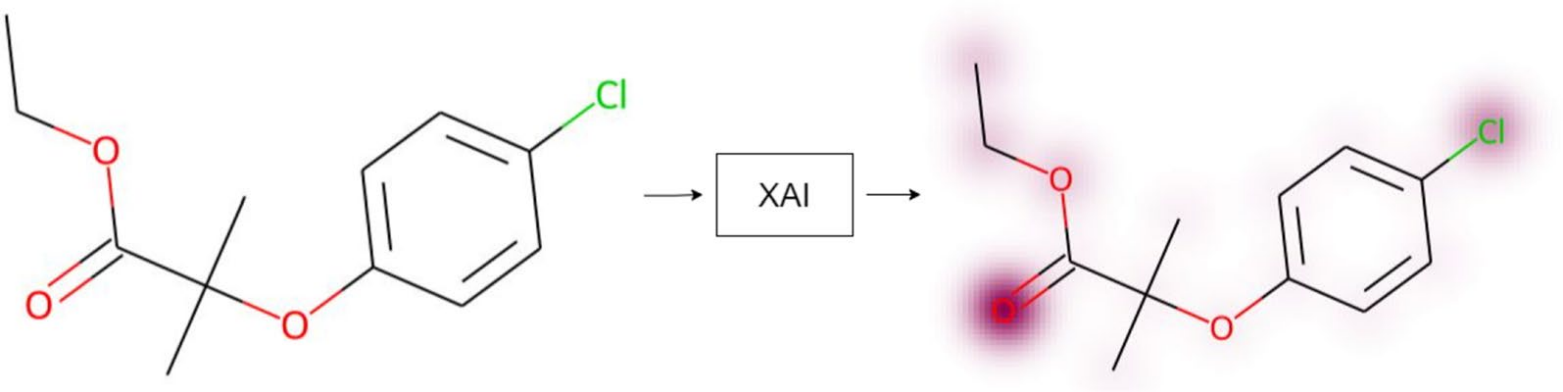
Data scientists create models that predict molecular properties and XAI reveals logic connecting substructures to the prediction: (left) a compound of interest is selected for inspection; (center) contributions for the predicted property of interest are calculated with an XAI method that delivers one score for each atom; (right) overlaying a molecular structure with those atom-scores

In chemistry, a visual approach to XAI involves visualizing atomic contributions to specific properties predicted by a model [5]. Figure 1 illustrates the process of generating explainability and overlaying a molecular structure with the information gained. Some atoms are highlighted, indicating that the model considers them important, which means that these atoms contribute more to the prediction than others. Such XAI visualizations can facilitate both the inclusion of domain experts in the development cycle and interaction with other experts and non-experts alike, for instance, when models are to be explained to regulatory agencies or when aiming to build trust in the results.

To support the analysis of large sets of compounds, cheminformatics tools allow users to explore the data by means of exploratory visualization techniques, for example by projecting a high-dimensional space into a low-dimensional space and enabling interactivity. One common desired outcome of multidimensional projection techniques is to preserve the relative distances between the samples as much as possible, either globally or in neighborhoods of similar entities [6–14]. By representing compounds in a two-dimensional space, the chemical space can be explored, and similar compounds can be identified [15–20].

Visualization-based cheminformatics tools are crucial in complex scenarios where data scientists and chemists analyze large sets of compounds and the output of AI models and XAI methods. Many goals in this context (e.g., to improve model accuracy) can be achieved by executing a series of abstract analytical tasks that will lead to data-driven decision-making. Each task can be carried out with the support of a variety of technologies, such as specific human-interaction and visualization techniques. Based on the experience acquired in our collaborations with data scientists and chemists, we identified three main tasks (Explore, Understand, and Compare) that help them to achieve their goals. For each task, we explain *why* it is relevant, give a few examples of *how* it can be performed, and relate it to use cases defined in this article in which it is a key element of the analysis:

- **Task Explore: Exploring chemical space** *Why*: to gain an overview of the entire dataset and explore compound neighborhoods; to select elements of interest, such as clusters and compounds; to find better ways of representing the chemical space, such as fingerprints, chemical properties, and the latent space of chemical models [21]. *How*: (a) users interact with an overview representation of the data and select interesting compounds for detailed inspection; (b) the dataset contains various types of compound representations, and users use each type to create projections that provide multiple perspectives on the chemical space. *Use cases*: 1, 2, and 3.
- **Task Understand: Understanding model behavior** *Why*: to understand why a model returns a particular prediction; to identify patterns correlated to good/poor predictions; to increase trust in the reported results; to check whether the model’s reasoning matches expert knowledge. *How*: (a) users select groups of compounds and compare the explanations extracted from a model; (b) explanations from a model are mapped to the various parts of a molecular structure, and users choose to validate whether the highlighted regions do, in fact, contribute to solubility. *Use case*: 1.
- **Task Compare: Comparing models and XAI methods** *Why*: to select or discard a model based on prediction performance, interpretability, or a trade-off between the two; to identify better XAI methods. *How*: (a) users have two models with similar accuracy and compare their explanations to select that which is more consistent with chemical knowledge; (b) users compare the predictions of two models and identify specific regions of the chemical space in which both models perform poorly; (c) users compare explanations from two XAI methods and identify agreements and disagreements. *Use case*: 2.

Tools with exploratory functionalities designed for chemical spaces  [19, 22–26] and molecular-representation methods [27–29] can be used for the purpose of *Task Explore*; tools that were not designed for chemical data, can also be used, but may limit the analysis.

*Task Understand* is addressed by a few approaches [30–32] that utilize various XAI methods to highlight contributions of compound substructures. In general, data scientists write scripts that visually map the explanations onto molecular diagrams, using functionalities from programming toolkits (e.g., a function from RDKit originally created for *similarity mapping* [33]). The resulting images are explored individually or in small portions in a non-interactive fashion.

*Task Compare* is a broader task, and many tools [34–38] help data scientists to find (dis-)similarities in prediction behavior, performance, training behavior, and interpretability of models to choose the most suitable model. The capabilities of these tools include comparison of models using performance metrics, model interpretability, or other architecture-specific measures. However, we did not find any interactive tool designed for chemistry tasks that combine visualization of performance metrics and model interpretability. Data scientists can use programming toolkits [39, 40] with analytical and visualization features to accomplish *Task Compare*. However, this approach is limited because interactive and coordinated visualizations cannot be promptly used out of the box.

In conclusion, while many of the defined tasks can—to some extent—be addressed by combining available tools, none enables integrated and interactive in-depth analysis of AI models and XAI methods. To close this gap, we propose CIME (ChemInformatics Model Explorer), an interactive web-based system that allows users to inspect model explanations, analyze models, and screen sets of compounds. CIME enables users to visualize explanations overlaid on chemical structures and to explore the chemical space through multidimensional projection. Our goal is to facilitate the communication between data scientists and chemists and to provide ways to compare and analyze chemical ML models by means of visualization of AI explanations and exploratory visualization techniques.

In the following two sections, we provide details about the implementation of CIME and demonstrate its use. In the Implementation section, we refer to *Task Explore*, *Task Understand*, and *Task Compare* whenever a feature of CIME is directly associated. In the Results section, we refer to the tasks by linking them to use cases in which their core ideas are achieved.

## Implementation

CIME is an extension of the ProjectionPathExplorer by Hinterreither et al [41]. The front-end of the application is a website written in TypeScript, and it uses the React framework [42]. Although the ProjectionPathExplorer web-application is standalone by default, providing all CIME-related features requires a back-end. We therefore developed a server-side Python application that uses the bottle framework [43] and can be accessed via a web-API (Application Programming Interface).

**Fig. 2:**
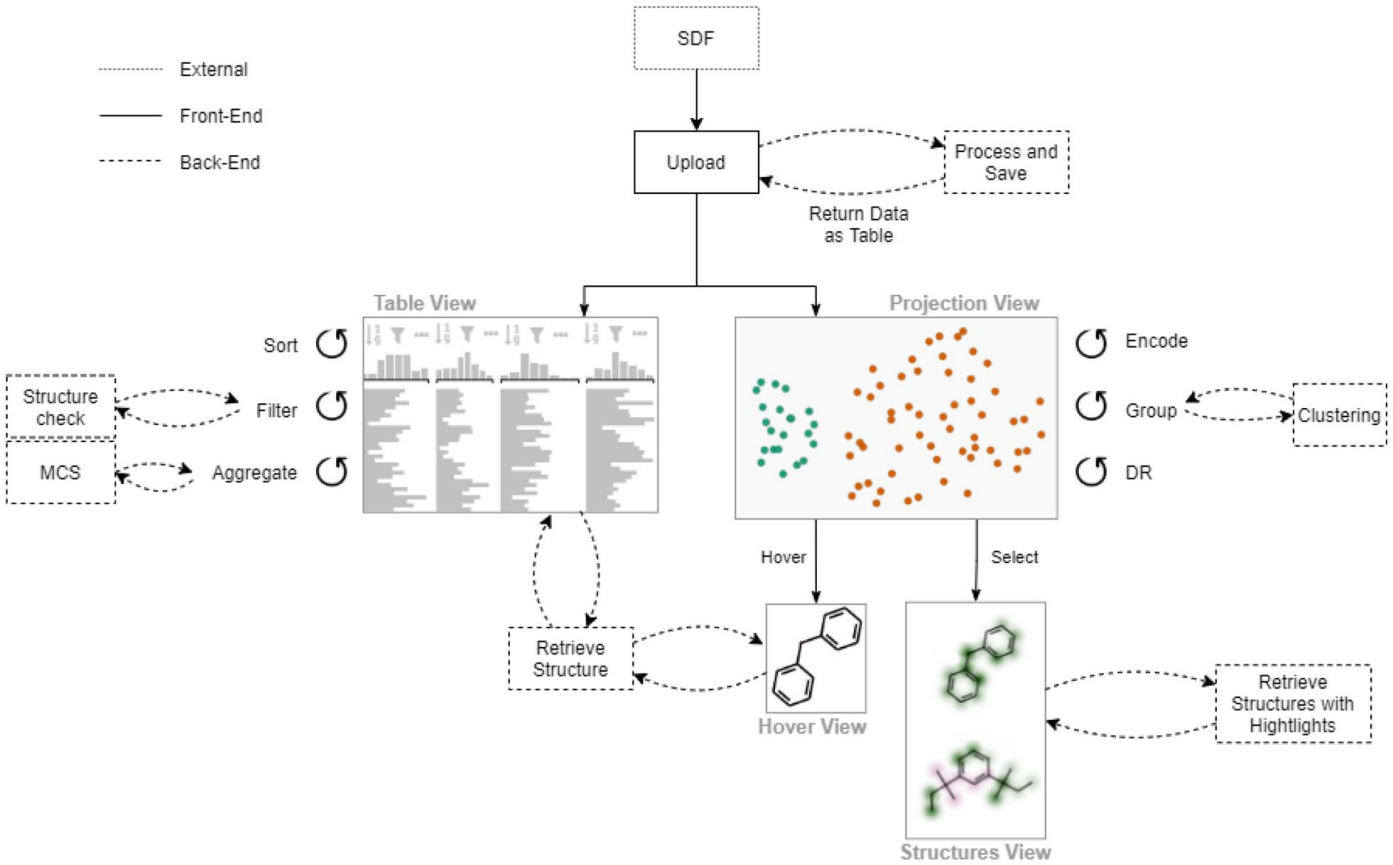
Workflow illustrating how users interact with the tool (solid line) and how the front-end web application communicates with the back-end server (dashed line). Creation of the SDF files is done externally (dotted box)

Figure 2 gives an overview of the interactions between users, front-end, and back-end.

Since chemists are familiar with Structure Data Format (SDF) files, and the format provides a clear structure of additional (atom-level) properties, we use them to define datasets of chemical compounds. The front-end, however, can only handle files in table format. The back-end is used to convert the provided SDF into the format required for the web application.

Furthermore, all features related to chemical compounds (substructure calculations, structure rendering, etc.) are accessed over the API by the front-end.

CIME is an open-source project hosted at github.com/jku-vds-lab/cime. In the following subsections, we provide more details about the implementation of CIME.

### Data processing

The following subsections detail how a suitable dataset is generated and how this dataset is transformed and augmented in the back-end, and describes various approaches to rendering chemical compounds.

#### SDF generation

To get started with the tool, users must generate a suitable SDF file that contains a set of chemical compounds of interest. For each compound, additional information can be provided, such as its molecular fingerprint, molecular properties and predictions, or coordinates of a predefined projection. If users do not provide fingerprint data, the system will calculate 256-bit Morgan Fingerprints [44] by default. For the fingerprint calculation, we fix the radius to 5 and do not use count values. Furthermore, users can specify attribution scores at the atom-level that were generated by an XAI method, or any other method, for instance, the Gasteiger Charges [45]. An example of how to create such a file can be found at github.com/jku-vds-lab/cime/tree/main/Examples. The SDF file is highly customizable to user needs (i.e., users can add any information of interest) and it is model-agnostic.

#### Data transformation

In the back-end, we use the RDKit Python library [40] to load the SDF file and iterate over the compounds in the dataset. For each compound, we derive its SMILES [46] string and extract its compound-level properties from the dataset (i.e., scalars or other values that are specified for the whole compound) to bring it in a tabular format. Properties that have a vector format, such as atom-level properties (i.e., properties that have one value for each atom in the compound) cannot be transformed into table format, since the vectors can have different lengths for each compound. To solve this problem, we serialize this kind of data and store it in a single additional column for later use. Depending on the size of the dataset, the initial data preparation can be time-consuming, as in many cases numerous compounds must be processed. However, once the dataset has been prepared, it is stored on the server and can be reused in later sessions.

#### Data augmentation

When the front-end requests a dataset from the back-end, the data is simplified and returned as a table. First, we remove the serialized column that contains all the information about atom-level properties, since it is not needed initially by the front-end. The column names of the dataset are then changed such that they include additional information that can be utilized in the front-end (e.g., specific columns—for example, those containing fingerprint data—belong together, but are spread across the whole table). Additionally, the tool checks whether fingerprints are provided in the dataset, and automatically adds default fingerprints otherwise.

#### Compound rendering

After dataset processing, one of the main tasks of the back-end is the rendering of two-dimensional compound structures. The back-end API provides a function that takes a SMILES string as input and returns an image of the two-dimensional structure of the compound. If a **list** of SMILES strings is provided, there are several ways of processing them:

- List of images: For each SMILES string in the list, we return a two-dimensional image of the compound structure.
- Single image: The maximum common substructure (MCS) of all compounds is calculated. An image of the two-dimensional MCS is returned.
- List of images with MCS highlight: The MCS of all compounds is calculated, and a list of images is returned with the MCS on the two-dimensional structure of each compound highlighted.
- List of images with contribution highlight: For each compound in the list, we retrieve the corresponding data point from the stored table. We extract the serialized column that contains the atom-level information and return images of the two-dimensional structure of the compounds with the attributions color-coded in green (positive score) and magenta (negative score). The magnitude of the value is displayed with contour lines.

The rendering of compounds and most compound calculations are done with the help of RDKit functions.

#### Clustering

The back-end has a function that calculates clusters of the provided data using HDBSCAN [47]. The API call takes as input a list of x and y coordinates, and custom hyperparameters.

### User interface

**Fig. 3:**
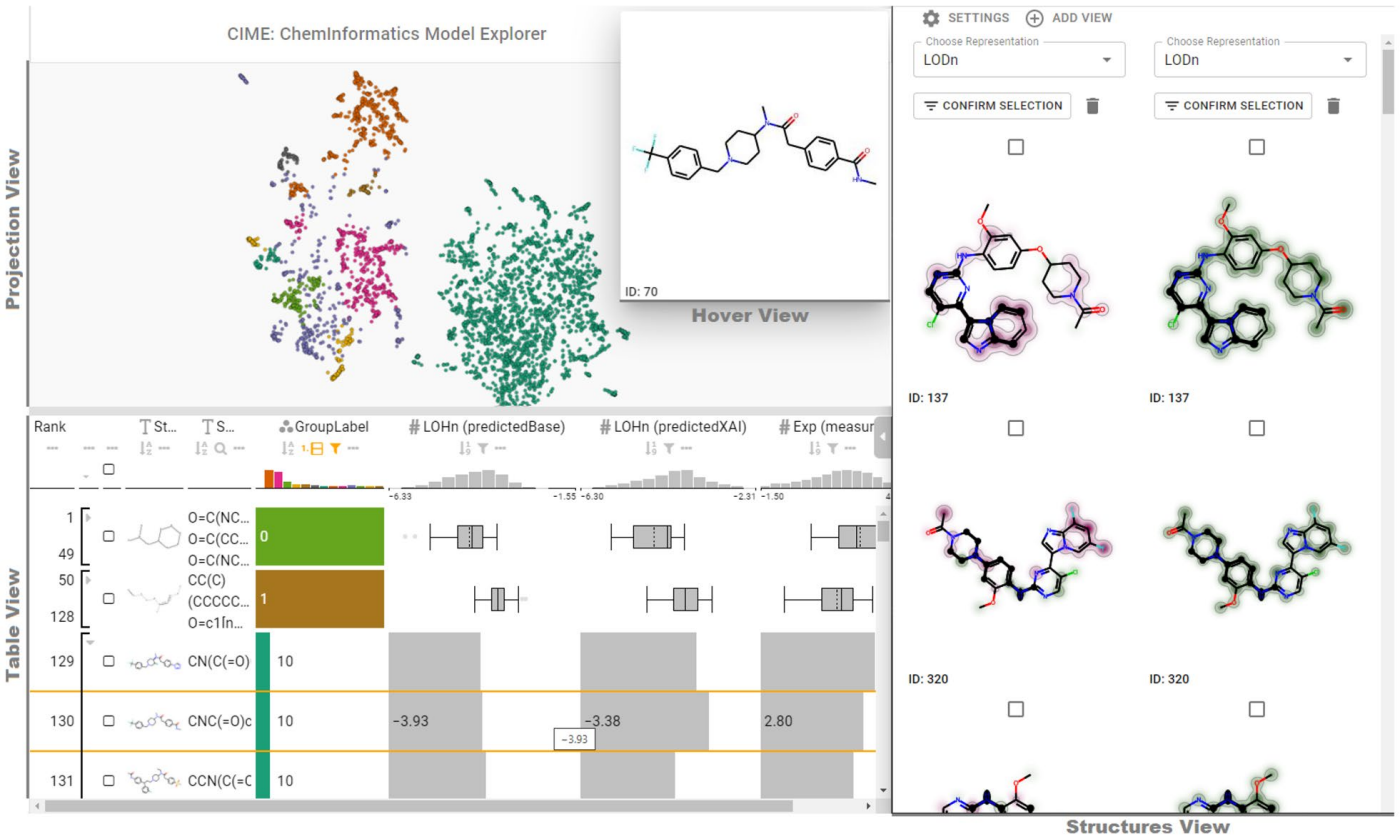
User interface of the CIME web application

Figure 3 shows the CIME front-end composed of four linked views: (1) the *Projection View*, which shows a scatterplot with the projected compounds, (2) the *Table View* for viewing and filtering information about the compounds, (3) the *Hover View*, which displays compound structures, and (4) the *Structures View*, which displays selected compounds and attributions. The following subsections provide details about these views and how users can interact with them. Figure 2 illustrates CIME’s workflow and how the front-end communicates with the back-end.

#### Projection view

Once users have uploaded a file, data points are shown in a two-dimensional scatterplot with random initial positions—if x and y coordinates are not explicitly provided—and can be projected using Uniform Manifold Approximation and Projection (UMAP, [48]) as dimensionality reduction (DR) technique. Users can choose the attributes that are to be used for projection and whether they are to be standardized to have a zero mean and unit variance. Fingerprints, latent space representations from neural networks, or molecular descriptors are good initial choices for the projection. An example of a projected dataset is shown in Fig. 3 “Projection View”. Projections can be stored, and users can switch between stored projections to compare different representations of the data (*Task Explore*).

To enable easier user interaction with the points in the scatterplot, the system offers a function for grouping neighboring points. Users can customize visual encodings of the points in the scatterplot. For example, the points can be sized by molecular weight or colored by group, as shown in the “Projection View” in Fig. 3. Grouping and interactively changing the visual encoding of data points help users to explore patterns and find clusters in the data (*Task Explore*). Using an encoding to visualize model performance metrics allows users to identify regions of the projection related to specific aspects of the model (*Task Understand*). For example, if dark colors represent inaccurate prediction, users can quickly identify groups of dark compounds, analyze them and check whether there are patterns that correlate to the inaccurate predictions.

#### Table view

By default, the data is projected to two dimensions and displayed in a scatterplot. To show all details of the original data, we include the well-established LineUp technique [49]. This additional view—which can be opened on demand — facilitates interactive filtering and exploration of the chemical space (*Task Explore*) and comparison of multiple models by various performance metrics (*Task Compare*). Users can filter the table by providing the SMILES string of a compound substructure, the back-end calculates whether the substructure is included in each of the compounds. The interactive table also allows users to group compounds and show summary visualizations of the data, as illustrated in Fig. 3 “Table View”. For the compound structure, the summary visualization is the maximum common substructure of the compounds.

#### Hover view

Users can hover over points in the scatterplot or rows in the LineUp table to show the 2D structure of the corresponding compound in a separate view, as illustrated in Fig. 3 “Hover View”. This feature helps users to quickly understand the nature of the compound (*Task Explore*).

#### Structures view

Selection of several data points prompts the tool to open a side view that shows a list of the corresponding chemical structures. The structures in this list highlight the maximum common substructure of all selected compounds and can also be aligned according to this substructure such that differences and similarities are better visible to users. In this view, users can choose from a list of attribution scores if they previously defined them in the SDF file. Analyzing model explanations helps users to better understand a model’s behavior (*Task Understand*). For the same compound, users can compare different attributions by means of additional views that are shown alongside each other. This can be helpful, for example, in comparing the explanations of multiple models (*Task Compare*), of different properties (*Task Understand*), or of different explanations retrieved from the same model using different methods. Further, users can manually filter the initial compound list to focus on the most interesting compounds. An example of the “Structures View” is shown in Fig. 3.

## Results

To give an idea of how to utilize CIME, we describe three use cases from authors of this paper, who are data scientists and computational chemists:

- Use case 1: Visualizing attributions to free hydration energy predictions using SHAP values.
- Use case 2: Comparing the attributions of models trained on a lipophilicity dataset.
- Use case 3: Comparing the latent space of a trained model to a fingerprint representation.

### **Use case 1**: visualizing attributions to free hydration energy predictions using SHAP values

In this use case, we explored the predictions of a model that was trained on the hydration free energy of a set of compounds. Hydration energy is one component in the quantitative analysis of solvation. It is a particular special case of water and describes the amount of energy released when one mole of ions is covered by water molecules. If the hydration energy is greater than the lattice energy, then the enthalpy of solution is negative (heat is released), otherwise it is positive (heat is absorbed). The more negative the hydration free energy, the more soluble in water the compound. Hydration free energy is an important physicochemical property to assess properties such as the bioavailability of small molecules.

With the goal of exploring the hydration free energy of compounds, we downloaded the Free Solvation Database (FreeSolv) dataset [50] which has already been used as a benchmark set in the past [51]. It consists of 642 compounds in the latest version along with their measured and calculated hydration free energy values. We then trained a CatBoost multiregression gradient-boosted tree model [52] to predict these variables. The features to train the model were the Morgan fingerprint count values [44] combined with MACCS keys [53]. The model performed well with an RMSE value of 1.03 as estimated by a 5-fold nested cross-validation approach (see Supplementary Material, Additional File 1 for details).

Aiming to understand how each atom contributed to the predicted hydration free energy value, we first calculated the tree SHAP (SHapley Additive exPlanations [54, 55]) values for every fingerprint feature. SHAP values are given in the same unit(s) as the target variable(s) — in our case hydration free energy—and indicate by how many units a feature pushed the prediction towards positive or negative values for a given instance.

**Fig. 4:**
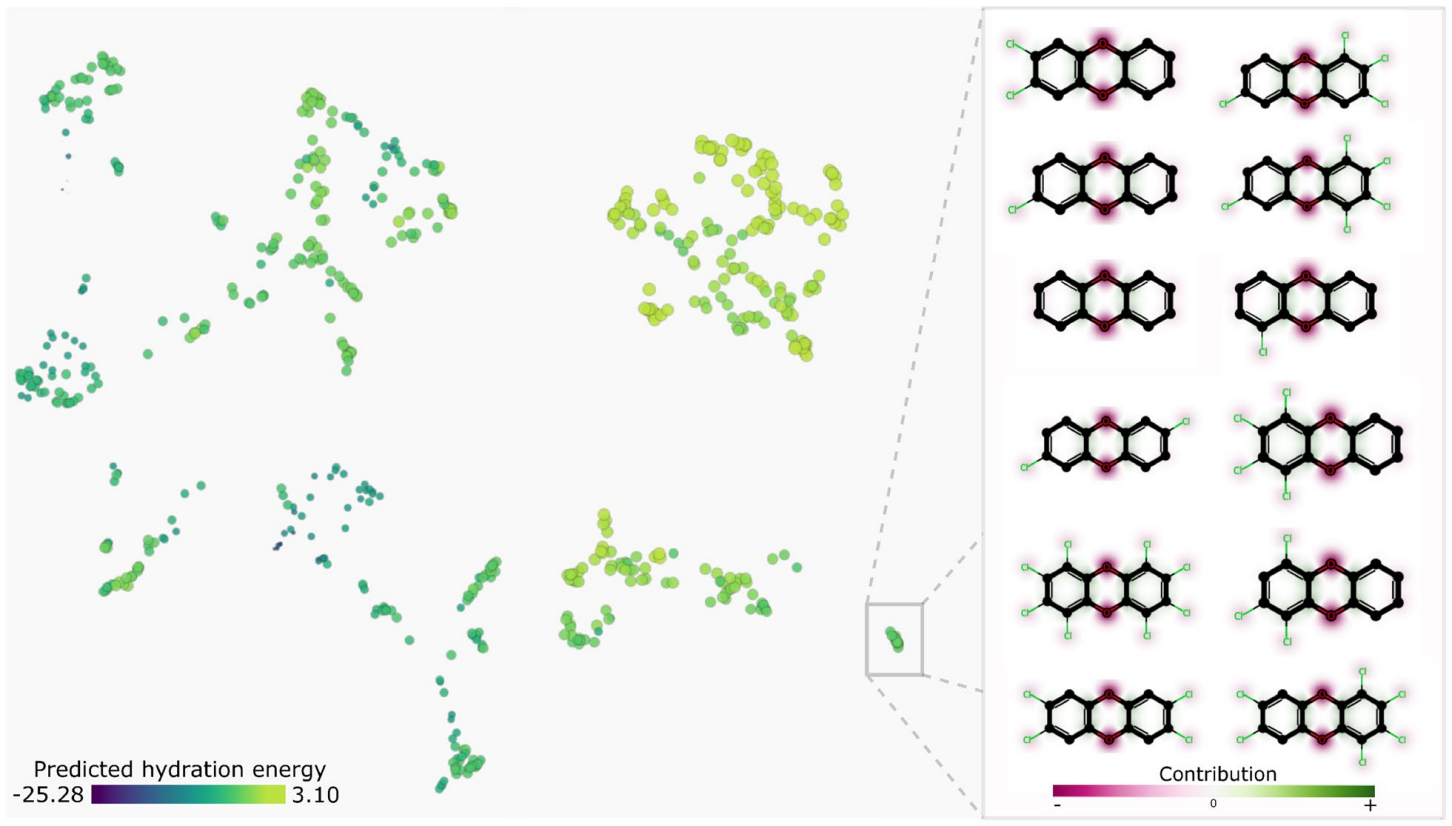
Compounds projected based on the SHAP values and colored by predicted free hydration energy. On the right, detailed view of a group and their maximum common substructure highlighted in bold

To analyze the chemical space, we derived a UMAP projection from the rank-based Spearman correlation matrix of the SHAP values of all observations. With this, we grouped the compounds by the similarity of the explanations (Fig. 4), making full use of the multivariate and feature interaction information. Which should be more expressive than just using Tanimoto similarity based on Morgan and MACCS fingerprints.

As we can see in Fig. 4, the projection reveals a few groups. The color indicates how nicely that SHAP values can be used to segregate compounds based on predicted hydration free energy of the trained model, since the segregation matches well the color diversion. The projection algorithm placed the compounds with positive predictions mostly at the top-right area. At the bottom-right, we found a group with 12 similar compounds in terms of structure and explanations, highlighted with the rectangle, and detailed on the right side of the figure. The bold stroke represents the maximum common substructure (i.e., the three rings that they have in common).

**Fig. 5:**
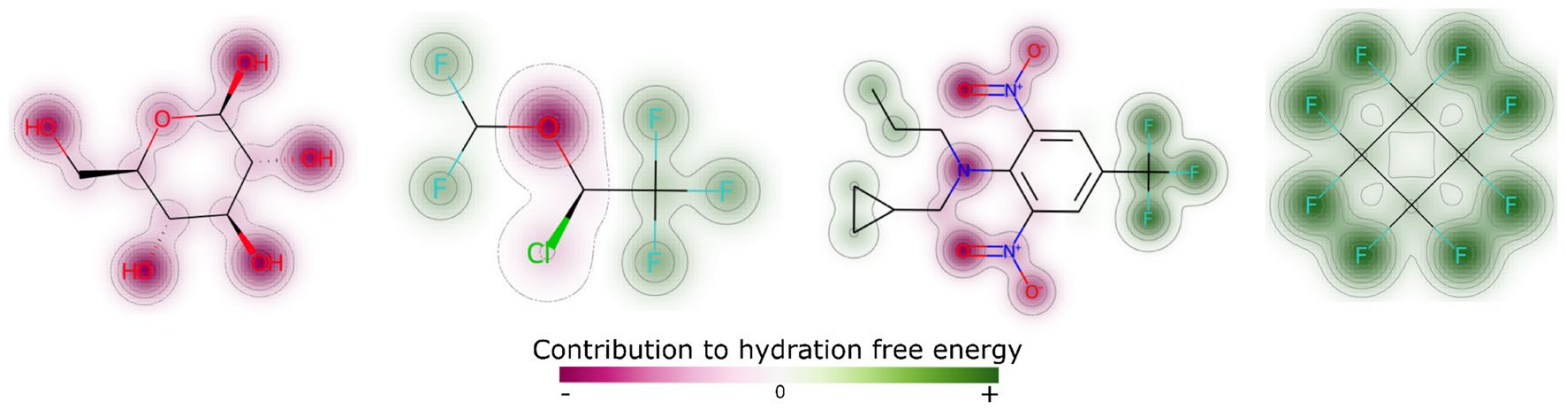
Four compounds and their atomic contributions to the prediction of hydration free energy. Magenta and green indicate contributions that decrease and increase energy, respectively

Furthermore, we used the SHAP values to understand how much each individual atom of a compound increased or decreased the predicted value. To this end, we determined for every non-zero feature the atoms that represent this feature, and then summed all SHAP values for every atom in the compound—these are our explanations, that indicate how each atom contributed to the prediction. As example, in Fig. 5, we show four compounds and how their atoms contribute to hydration free energy. For these compounds the less polar hydrocarbon regions appear in green, whereas polar atoms forming hydrogen bonds appear in magenta, as we would expect.

In this use case, we demonstrated how a set of molecules can be explored under the perspective of SHAP values (*Task Explore*). Exploring the chemical space considering how a model sees the data can help users to identify interesting groups of compounds. SHAP-based explanations allowed us to confirm that the model seems to identify which regions of the selected compounds contribute positively, and negatively, to hydration free energy (*Task Understand*).

### **Use case 2**: comparing the attributions of models trained on physico-chemical properties

Lipophilicity is an important parameter in medicinal chemistry, related to the pharmacokinetic properties of a drug [56]. Therefore, it is of great interest to monitor such property in drug discovery projects. Here, we explore a set of compounds examining their lipophilicity and compare two in-house models as for their interpretability.

The lipophilicity dataset was taken from the MoleculeNet datasets [57]. Two in-house pre-trained graph convolutional models (see [58] for more details on the training datasets) were used to predict logD of the compounds from the lipophilicity dataset. Here, LogD is the logarithm of the partition coefficient of a compound between octanol and water, taking into account the charge state of the compound at a physiologically relevant pH. The first model is hereafter referred to as the “base model”. The second model, here identified as “XAI model”, was designed to be more interpretable by adding constraints during training [59]. The dataset of 4200 compounds was uploaded to CIME. It contains the measured lipophilicity, the logD predictions by the two models, the models’ latent space representations and atom contributions for both predictions. The Class Attribution Maps (CAM) methodology was adapted to graph neural networks [30] to obtain the atom contributions for the two models.

Once the data had been uploaded, a UMAP projection was calculated based on the explainable model’s latent space representations. We then proceeded to explore different groups, the predictions obtained by the models and the related explanations. Here we present our findings related to one specific group that contains 26 compounds with high structural similarity (see Supplementary Material, Additional File 1 for a detailed view of the group and projection).

**Fig. 6:**
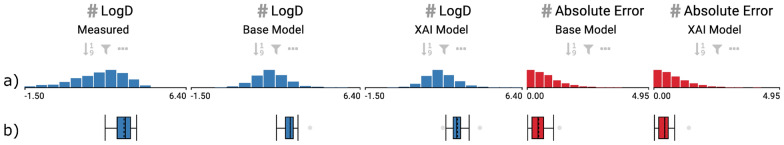
Screenshot from the LineUp table in CIME showing the predicted and measured logD values, and absolute error from each model as follows: a) histograms of values from the entire dataset; b) box plots of values from the studied group

Using CIME’s “Table View”, we display in Fig. 6 an overview of the measured and predicted logD and absolute errors from each model for the entire dataset (a) and selected group (b). We observe that for some compounds the predictions (of one or both models) are good with an error below 0.5 log units while others have predictions a bit off (errors above 0.5 log units)—see Supplementary Material, Additional File 1.

**Fig. 7:**
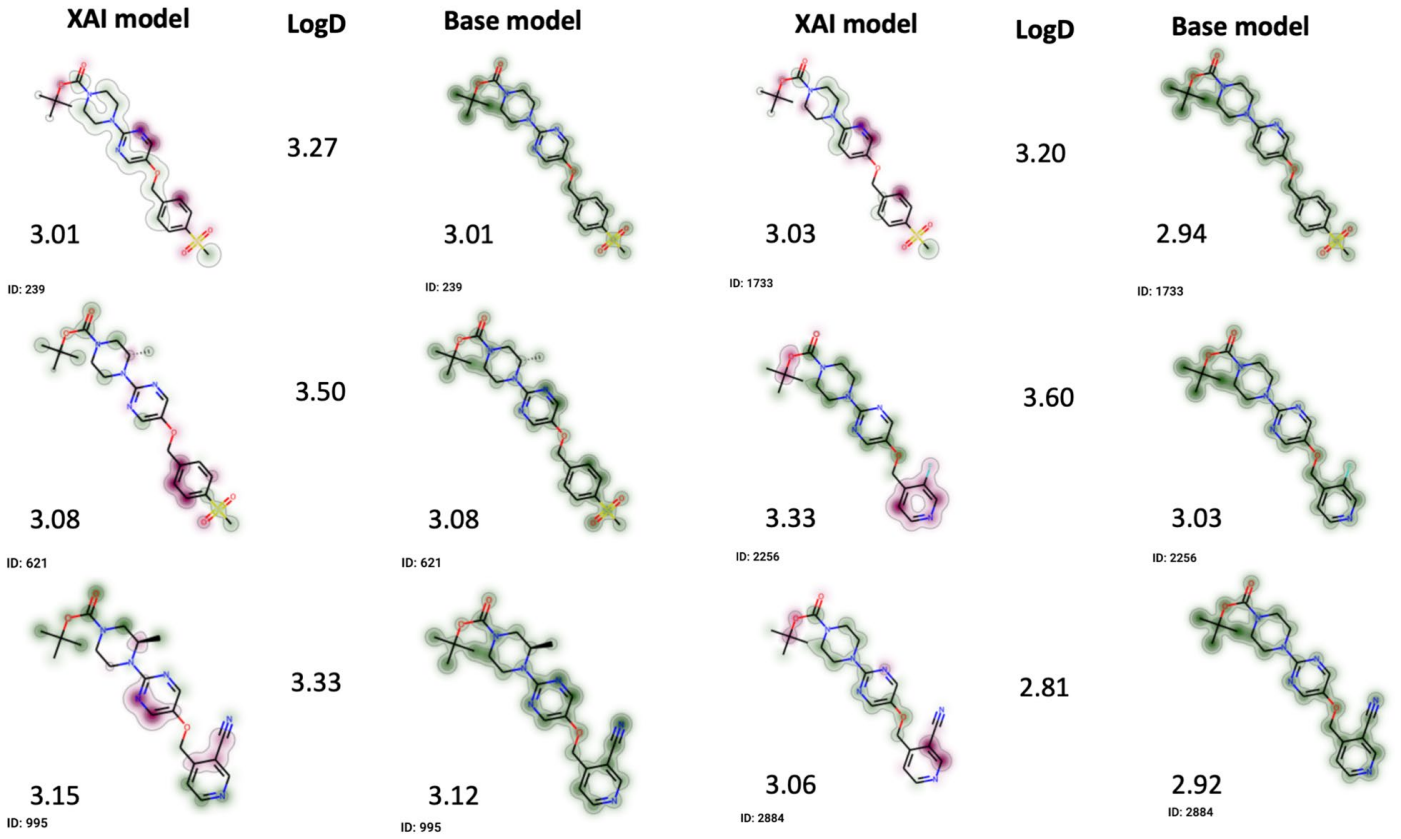
Comparison of attributions and predictions for the two models of interest (XAI and base model) for six compounds with low prediction error. The logD column reports experimentally determined lipophilicity. The number next to the compound structure corresponds to the model’s prediction. Magenta highlights correspond to atoms which are lowering the logD prediction, green highlights correspond to atoms which are increasing the logD prediction.

Figure 7 shows attributions from both models for a subset of accurately predicted compounds in the selected group. Note that magenta atom contributions are sites which push the prediction towards lower values of logD (i.e., less lipophilic), and green contributions indicate sites that push the predictions towards higher values of logD (i.e., more lipophilic). We observe that the attributions produced by the base model are uniformly green for all compounds, which is not useful to a chemist trying to find optimal positions for modifications. This is the case for all compounds of the cluster, not only for those shown in Fig. 7. Furthermore, the atom contributions according to the XAI model are more diverse and sparse: there are atom contributions labeled as (i) increasing lipophilicity, (ii) decreasing lipophilicity and (iii) as largely irrelevant to the prediction.

Both models give similar predictions.

In four out of six cases, the XAI model attributes lower lipophilicity to the ester group. Similarly, the heteroatoms in the three rings of the scaffold are often marked as lowering the lipophilicity, or at least are excluded from the green highlights. Both of which accords with a medicinal chemist’s intuition. Nevertheless, the attributions are far from perfect, especially from a stability point of view: some very similar compounds have different attributions in the XAI model (for example, molecules 239 and 621 only differ by one methyl group but have very different explanations).

This use case demonstrated how CIME can be used to compare attributions from two models (*Task Compare*) through the exploration of a test dataset (*Task Explore*), and might increase user trust in predictions made by an interpretable model. A similar workflow could be used for comparing two (or more) attribution methods for a single model; or one attribution method and one ground truth attribution in cases where ground truth explanations are known.

### **Use case 3**: comparing the latent space of a trained model to a fingerprint representation

Protein kinases feature prominently in the human genome [60], and kinase inhibitors are of particular interest in drug discovery [61]. Recently, Sydow et al. [62] have developed a fragment-library approach to generating novel kinase inhibitors. In this approach, known kinase inhibitors are split into smaller molecular fragments, and those fragments are then virtually recombined. While theoretically the number of potential new kinase inhibitors is limited only by the number of possible fragment combinations, in practice some of these “recombined” compounds will be more desirable than others, for instance, because of their physicochemical properties or synthetic feasibility. It is thus of interest to explore the large set of virtually generated candidates to find subsets of promising candidate kinase inhibitors.

Extended connectivity fingerprints (ECFPs) [63] are commonly used descriptors in ligand-based virtual screening. However, ECFPs encode only structural information. More abstract encodings pertaining to the prediction of physicochemical properties can be better expressed using latent space representations generated from deep learning models (i.e., replacing use of fingerprints with latent space representations to generate a projection). In this use case, we used the same in-house pre-trained explainable model as in Use Case 2 to generate the learned embeddings for the compounds and fragments in the kinase dataset.

**Fig. 8:**
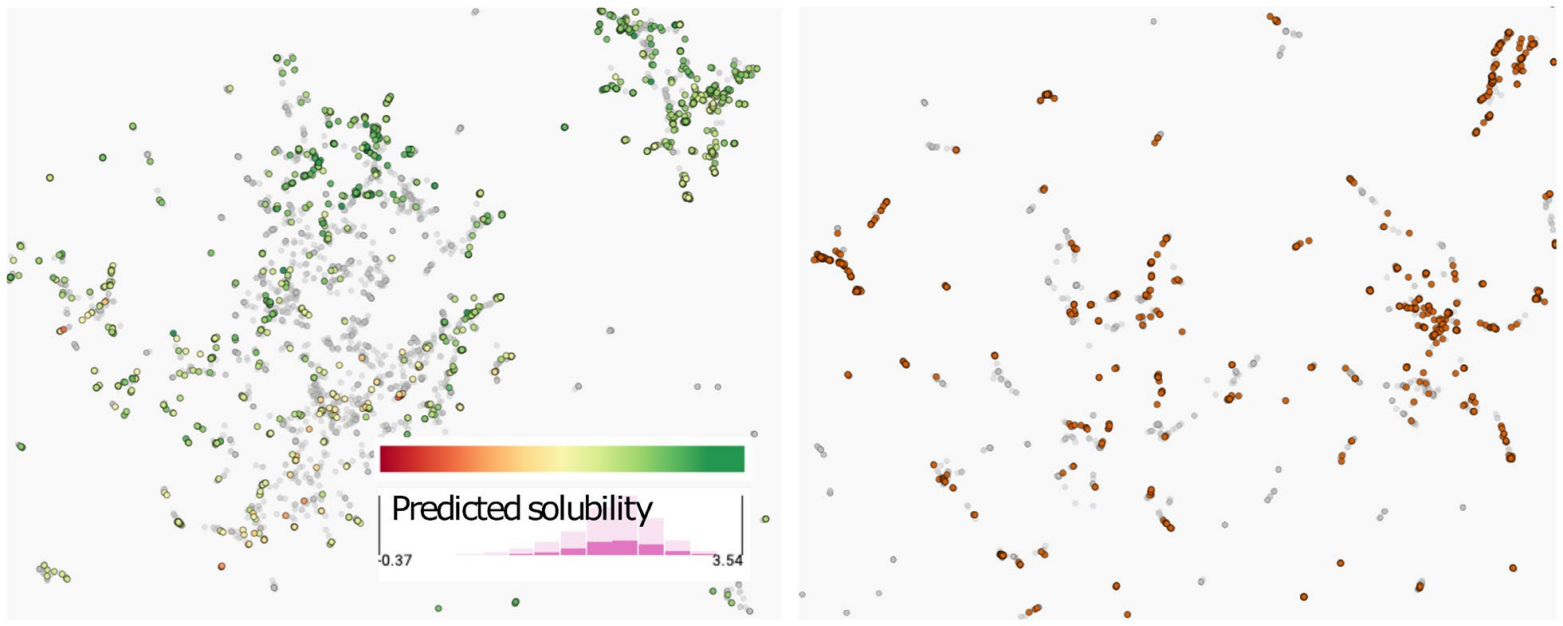
UMAP projection of kinase inhibitor *fragments*. Colored points correspond to fragments found in molecules that bind in the front pocket. Gray points correspond to fragments found in molecules that bind in other kinase pockets. Left: projection based on the latent space generated by a deep learning model, colored according to the predicted solubility. Right: projection based on the ECFP fingerprint representation.

In Fig. 8, we illustrate the representation of the *fragments* for both the latent space from a deep learning model (left) and the ECFP4 fingerprint (right). We highlight and color only the fragments known to bind to the FP subpocket. Regarding the positioning of the fragments, the visualizations suggest that the latent space generates a smoother representation compared to the ECFP4 fingerprint space. This makes intuitive sense since ECFP4 is a 2048-dimensional bitwise fingerprint based fully on structural features, whereas the deep learning representation is a 256-dimensional continuous vector. In the left part of Fig. 8, we colored the fragments by the predicted solubility and see that most of them are predicted to be soluble (i.e., they are between yellow and green). The fact that the analyzed “front pocket”fragments have generally higher predicted solubility is congruent with chemical rationalizations given in [62]. Since the ECFP4 fingerprint is not by itself predictive, we only highlight whether the compound is found in the front pocket or not in Fig. 8 (right).

Sydow et al. [62] provided a *recombined ligand* library of over 6 million potential kinase inhibitors, helpfully scoring the ligands based on their closest chemical similarity to compounds found in the ChEMBL database [64, 65], as measured by the Tanimoto similarity. By using this information, we can quickly identify regions in a projection where the recombined compounds are similar to *known molecules*.

**Fig. 9:**
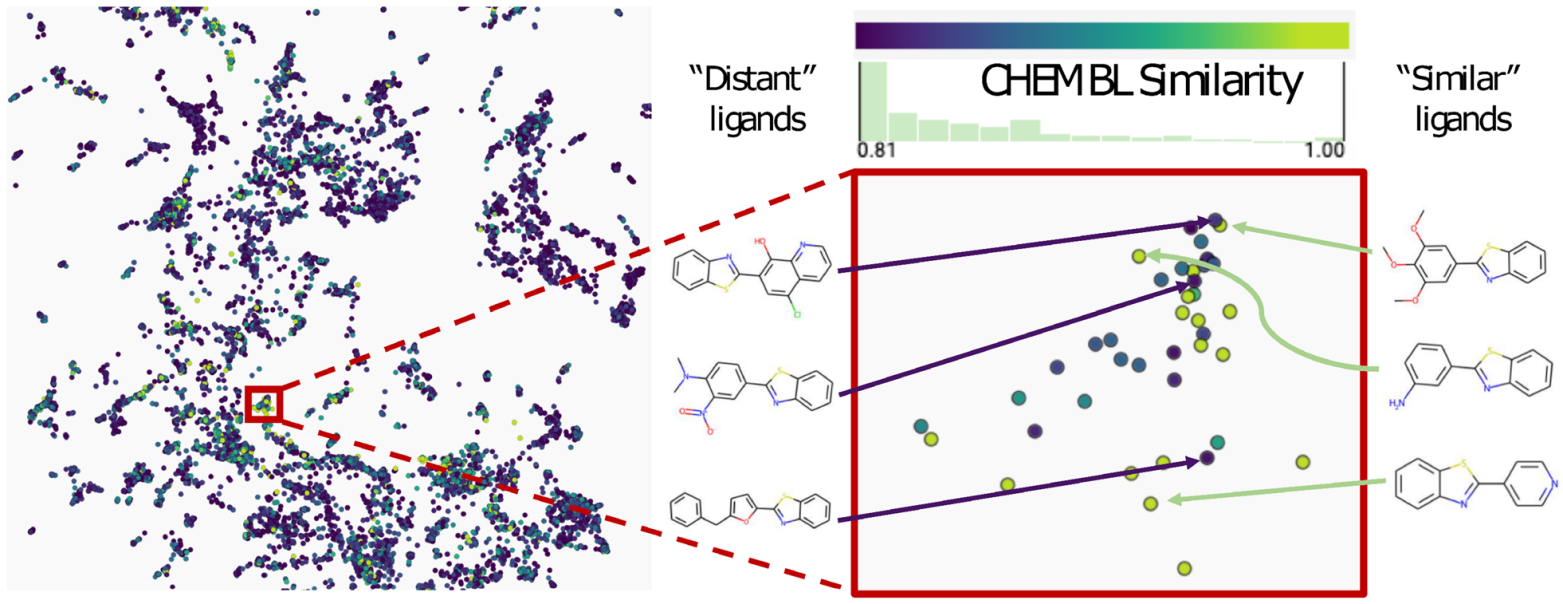
Visualizations of kinase inhibitors. Left: UMAP projection based on the latent space of recombined ligands with a Tanimoto similarity greater than 0.8 to at least one known ligand in ChEMBL. Ligands are shaded according to their maximum similarity to known ligands. Right: a region from the projection

We therefore projected the recombined ligands based on the latent space from a deep learning model, as was done for fragments in Fig. 8 left. We utilized only ligands with a Tanimoto similarity greater than 0.8 to at least one ligand in ChEMBL. Then, we colored the compounds according to their similarity to known ligands in ChEMBL (Fig. 9). This view of the recombined ligand space allows focusing on specific regions that are densely populated in compounds highly similar to existing compounds. The selected region is enlarged for a closer view, and several relevant chemical structures are revealed. We speculate that compounds that are different from the known ChEMBL molecules (“Distant ligands” in Fig. 9) but positioned closer to more ChEMBL-similar molecules in the fingerprint space are more likely to represent promising ligands than recombined molecules that are in dark blue regions (none of their neighbors is close to a known molecule).

This use case demonstrated how CIME can be utilized to explore a chemical space and to compare molecular representations for a set of labeled compounds (*Task Explore*). By using an approach based on exploring two types of similarities, we showed how CIME can be used to select smaller sets of pertinent candidate compounds from a large chemical space.

### Performance

**Fig. 10:**
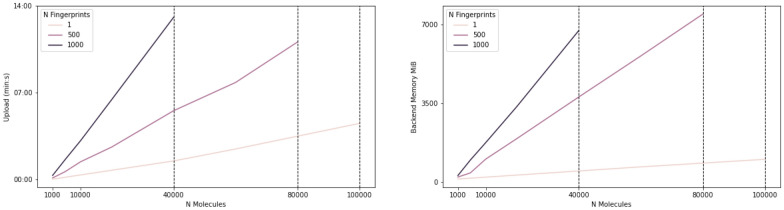
The line-charts show the loading time (left) and the memory usage of the backend (right) for datasets with increasing number of compounds. Color indicates the number of fingerprints provided in the dataset. The vertical dashed lines indicate the limitations of the system w.r.t. the number of fingerprints

We conducted structured benchmarks on two different machines by gradually increasing (i) the number of compounds in the dataset and (ii) the number of features used for projection (i.e., fingerprints). A summary of the benchmark is visualized in Fig. 10. We provide a detailed description of the CIME benchmark in the Supplementary Material, Additional File 1.

Overall, CIME dealt well with datasets of up to 20,000 compounds and 1,000 fingerprints. Beyond these thresholds, we experienced longer loading times (i.e.,>= 5 minutes). The results are better if fingerprints are not handled by the system; that is, the projection is precalculated and stored in the SDF. Not having fingerprints uploaded or computed by CIME resulted in a considerable drop in memory usage in both back- and front-end. We tested datasets of up to 100,000 compounds with only 1 fingerprint to simulate this scenario in our benchmark, where CIME generally handled the datasets well, with only LineUp’s initial loading being slow at 5-20 seconds when over 60,000 compounds were used.

### Future work

Currently, the tool does not allow direct comparison of different projected spaces: users see only one projection at a time. However, we are working on a feature that allows displaying two projections next to each other for better comparison of representations.

Another limitation of the tool is its inability to save its current state, which means that users must show their live analyses directly to collaborators or make screenshots to document the results. We are working on a solution that simplifies collaboration between users on different devices and enables users to store their analysis and continue it at a later point.

CIME enables users to select compounds and display each compound structure overlaid with attributions. Although CIME allows users to show structure-based aggregations of selected compounds using MCS, it is not possible to display aggregations of attributions of a list of compounds. We are not aware of existing visualization techniques that are capable of displaying multiple weights (attributions) per atom effectively.

Regarding the visual representation of compounds, users can neither interact with the compounds nor check the numerical values of atom contributions. However, we plan to adapt a JavaScript library for drawing the compounds in the front-end and make them interactive.

Currently, only one algorithm is available for projecting and one for clustering data—UMAP and HDBSCAN, respectively. Users can alternatively include precalculated projections and cluster affiliations in the SDF file. CIME can also be enhanced programmatically by users to include additional projection methods. As part of future work, we plan to provide more projection and clustering algorithms directly within the tool. However, not every library can be integrated into CIME’s official repository due to licensing restrictions

## Conclusion

We have presented the ChemInformatics Model Explorer (CIME), which facilitates work with data from chemical compounds, AI models, and XAI methods. CIME is a significant step towards a better understanding and comparison of AI models in the chemical domain. It enables users to interactively explore chemical spaces by combining overview and detailed visualization techniques. CIME’s model-agnostic nature allows it to be applied to a variety of cheminformatics tasks, as demonstrated in three use cases involving domain experts. We believe that CIME improves collaboration between chemists and data scientists and thus helps to improve cheminformatics workflows.

## Availability and requirements

Project name: CIME–ChemInformatics Model Explorer

Article project version: cimeV0.1.20

Project home page: github.com/jku-vds-lab/cime

Demo: cime-demo.jku-vds-lab.at

Operating systems: Platform-independent

Programming language: TypeScript, Python

Other requirements: the front-end runs on Chrome 95.0+, Edge 84.0+, Firefox 94.0+, or Safari 15.1+ web browsers; the back-end requires Python 3.8.5, RDKit 2020.09.5, bottle 0.12.18, hdbscan 0.8.27, joblib 0.17.0, and bottle-beaker 0.1.3.

License: BSD 3-Clause License.

## Supplementary Information

**Additional file 1**. Supplementary Material including details about the benchmark and use cases.

## Data Availability

We modified publicly available datasets by adding information extracted from AI models and XAI methods for the exclusive purpose of demonstrating the tool in this article. The AI and XAI methods used to modify the datasets are not part of CIME, and therefore beyond the scope of this work. However, we provide a Python script that gives an example of how users can create their datasets to use with CIME at github.com/jku-vds-lab/cime/tree/main/Examples. The *original* datasets for use cases 2 and 3 (i.e., without AI and XAI data) are open and freely available under MIT license (github.com/deepchem/moleculenet, github.com/volkamerlab/KinFragLib). The “FreeSolv” dataset for use case 1 is available at escholarship.org/uc/item/6sd403pz (version 0.51) under CC BY-NC-SA 4.0 license. The derived datasets that we utilize in the use cases (i.e., with AI and XAI data) are available at www.doi.org/10.17605/OSF.IO/KNS6M under the following licenses: use cases 2 and 3, CC BY 4.0 Attribution license (creativecommons.org/licenses/by/4.0/); and use case 1, CC BY-NC-SA 4.0 (creativecommons.org/licenses/by-nc-sa/4.0/). These datasets were not used during the development of CIME and are not part of the system. They are not published in CIME’s git repository. The datasets can be downloaded from the data-repository and explored with CIME through the DEMO webpage, which is hosted and maintained by JKU Linz, without any commercial interest.

## Abbreviations

AI: Artificial intelligence
API: Application programming interface
CAM: Class Activation Maps
CIME: ChemInformatics Model Explorer
DR: Dimensionality Reduction
ECFP: Extended connectivity fingerprint
HDBSCAN: Hierarchical density-based spatial clustering of applications with noise
MCS: Maximum common substructure
ML: Machine learning
QSAR: Quantitative structure-active relationship
SDF: Structure data format
SHAP: Shapley additive explanations
SMILES: Simplified molecular input line entry system
UMAP: Uniform manifold approximation and projection
XAI: Explainable AI
