# Supplementary material for: ChemInformatics Model Explorer (CIME): exploratory analysis of chemical model explanations
Source: J Cheminform. 2022 Apr 4;14:21. doi: 10.1186/s13321-022-00600-z (PMC8981840; doi:10.1186/s13321-022-00600-z)
Supplement: Supplementary file 1 — Additional file 1. Supplementary Material including details about the benchmark and use cases. [file 13321_2022_600_MOESM1_ESM.pdf]

Supplementary Materials

ChemInformatics Model Explorer (CIME): Exploratory analysis of chemical model explanations

Christina Humer, Henry Heberle, Floriane Montanari,
Thomas Wolf, Florian Huber, Ryan Henderson, Julian Heinrich and Marc Streit

julian.heinrich@bayer.com, marc.streit@jku.at

March 21, 2022

Memory usage and response time

Although CIME was designed to handle up to 20,000 compounds and around 1000 fingerprints, users can upload larger datasets. While the projection algorithm uses fingerprints and consumes much CPU while running in the browser, memory is a bottleneck in the back-end due to storage and processing of SDF. Depending on where the back- and front-end run, computational power will define the limits of the tool.

To understand CIME's limits we ran a benchmark with two computers. They are laptops with specifications defined in Table 1.

Table 1: Specification of computers used in the benchmark.

**Computer**	**OS**	**Processor**			**RAM**	**GPU**
		**Model**	**Frequency**	**Cores**		
A	macOS 11	Intel Core i7-9750H	2.6 GHz	6	32 GB	AMD Radeon Pro 5300M 4 GB
B	Windows 10	Intel Core i7-10750H	2.6 GHz	6	16 GB	NVIDIA GeForce RTX 2070 Max-Q

To run the benchmark, we created bigger datasets by replicating the compounds into datasets with up to $200,000$ and uploaded them to the system. The number of fingerprints were also explored, in the range of 1, 500, and 1000 fingerprints. Explainability, properties and predictions were fixed to 25. We ran the back-end using Docker 20.10.7 (docker.com) and accessed the front-end using the web browser Google Chrome 95 (google.com/chrome).

The benchmark consisted of uploading a dataset, loading all compounds into LineUp, executing the UMAP projection using fingerprints, and interacting with the graphic user interface to check if it is responsive, using CIME version 0.1.19. We restarted the Docker container whenever a new dataset was uploaded.

Table 2 reports the relation between dataset dimensions and the columns: (Upload) the time that the backend took to process the upload of a dataset; (RAM) amount of memory used in the

backend just after the upload completed in MiB; (LU) whether or not LineUp was able to load all items of the dataset; (UMAP) the time to compute the projection in minutes:seconds (MM:SS). We highlight in gray the upper-limits of our tests that worked in both computers. Asterisks (*) indicate when CIME stopped working.

Table 2 shows that in many cases we concluded the upload successfully, however we couldn't complete the UMAP projection. This might be connected to the fact that we are running both front- and back-end in the same computer and a high amount of memory is used in the backend, limiting the resources available for the browser, where LineUp is populated with compounds and the projection is computed. Memory is used in the browser: (a) to store fingerprints and compute and store a distance matrix for the projection with size NxN, where N is the number of compounds; (b) to store compounds and their properties in another matrix in the LineUp object. Therefore, we expect that the limits of CIME in computer A and B would increase if the back-end is running on another computer with enough RAM, i.e., 2GB available for a dataset with 100,000 compounds and 1 fingerprint, or 10 GB for a dataset with 100,000 compounds and 500 fingerprints or 60,000 compounds and 1,000 fingerprints.

Table 2: Benchmark table showing number of compounds (column Compounds), the number of fingerprints (column Fin.), time to complete the upload of a dataset (column Upload), the amount of memory used in the back-end in MiB (column RAM), whether the LineUp table worked or not (column LU), and the time to complete a multidimensional projection (column UMAP) in minutes:seconds (MM:SS). An asterisk (*) indicates that the task could not be completed. After uploading the dataset, we loaded all compounds into LineUp and executed the UMAP on fingerprints without normalization. Lines in gray indicate the upper-limits of our tests that worked in both computers.

**Dataset**		**Computer A**				**Computer B**			
**Compounds**	**Fin.**	**Upload**	**RAM**	**LU**	**UMAP**	**Upload**	**RAM**	**LU**	**UMAP**
1,000	1	00:01	124	✓	precomputed			✓	
40,000	1	01:29	482	✓	precomputed			✓	
60,000	1	02:27	664	✓	precomputed			✓	
100,000	1	04:31	1000	✓	precomputed	03:40	1100	✓	precomputed
200,000	1	*	*	*	precomputed	*	*	*	precomputed
1,000	500	00:07	210	✓	00:04			✓	
5,000	500	00:37	394	✓	00:24			✓	
10,000	500	01:25	1013	✓	00:43			✓	
20,000	500	02:36	1921	✓	01:25			✓	
40,000	500	05:32	3777	✓	03:02	04:45	3800	✓	02:45
60,000	500	07:49	5604	✓	*	07:03	5500	✓	05:00
80,000	500	11:05	7467	✓	*	10:04	7500	✓	*
100,000	500	*	*	*	*				
1,000	1000	00:19	278	✓	00:05			✓	
5,000	1000	01:35	966	✓	00:28			✓	
10,000	1000	03:07	1748	✓	00:54			✓	
20,000	1000	06:25	3348	✓	01:53	04:59	3400	✓	01:45
40,000	1000	13:04	6737	✓	*	10:42	6600	✓	*
60,000	1000	*	*	*	*	*	*	*	

Overall, CIME easily handled datasets with up to $20,000$ compounds. For a higher number of compounds, we recommend pre-calculating the projection and adding only one fingerprint to the SDF file. This will drastically reduce memory consumption and increase speed response time in the browser. In the current version, adding 1 fingerprint to the SDF will make CIME ignore the calculation of fingerprints (default behavior when CIME does not find fingerprints in the dataset). Users must add instead 1-bit fingerprint to their SDF and provide the x,y coordinates. Future versions of CIME should not require the 1-bit fingerprint and calculate fingerprints on request.

We are changing CIME's architecture and expect to have an improved version of the system by mid-2022. Please check the updated documentation in the git repository: github.com/jku-vds-lab/cime.

1 Chemical Calculations

We utilize various functions from the RDKit **??** framework. The following is a list of important RDKit functions used in CIME. We explicitly indicate those parameters that were changed from their default value:

- **Fingerprint Calculation**: calculated with RDKit's "GetMorganFingerprintAsBitVect" function; parameters are set to *(radius = 5, nBits = 256)*
- **Compound Image Rendering**: rendered with RDKit's "MolToImage" function
- **MCS - Maximum Common Substructure**: calculated with RDKit's "FindMCS" function; parameters are set to *(timeout = 60, matchValences = False, ringMatchesRingOnly = True, completeRingsOnly = True, atomCompare = AtomCompare.CompareAny, bondCompare = BondCompare.CompareAny)*
- **Highlight Substructure on Compound**: rendered with RDKit's "PrepareAndDrawMolecule" function
- **Contribution Highlighting**: rendered with RDKit's "GetSimilarityMapFromWeights" function; the following parameters can be changed by users in the front-end: size, contourLines, scale, sigma
- **Compound Alignment**: calculated with RDKit's "AlignMolToTemplate2D" function with *clearConfs = True*

Use cases

In this section, we bring supplementary information for the use cases:

- Use case 1: Visualizing attributions to hydration energy predictions using SHAP values.
- Use case 2: Comparing the attributions of models trained on a lipophilicity dataset.

Use case 1: Visualizing attributions to hydration energy predictions using SHAP values

ML modeling A nested 5-fold cross-validation was performed to identify the error and optimize hyperparameters. We obtained an RMSE of 1.03 on the predicted experimental values. To define the folds of the cross-validation in a way that very similar compounds are placed together in the same fold, we used hierarchical clustering (average linkage) to group them. Groups of compounds with a Tanimoto similarity of at least 0.75 were placed in the same fold. These folds define the outer-loop of the nested cross-validation. For each of the outer folds, an inner-loop of cross-validation was used to optimize the CatBoost hyperparameters using Optuna [1].

Use case 2: Comparing the attributions of models trained on a lipophilicity dataset

Multidimensional projection The projection was calculated using UMAP and the "fingerprint-XAI" properties of the compounds that are shown in the Projection Menu when using CIME with the provided dataset (See Figure 1).

Absolute error To identify areas of the chemical space where both models performed well, we calculated for each compound the *absolute error* [2] of predictions from the base and XAI models against the measure LogD. Therefore, each compound has two errors associated: base error and XAI error. We show the compounds colored by the mean value between their base and XAI errors in Figure 1 — approach used to identify a cluster with low error in both models. We see in the projection that most compounds have error much closer to 0 than to 4 (see color legend). Very few regions (dark) reveal extensive disagreement among predictions and experimentally measured logD values.

Histograms of predictions and measured LogD In Figure 3 we can analyze a summary of models' predictions and performance for the entire dataset with the histograms displayed at the top. Histograms in red (Base and XAI error) and brown (mean between Base and XAI errors) confirm that the errors from both models are closer to 0, i.e., they are left-skewed. Histograms in blue (measured and predicted LogD) show that the predictions' distributions are similar to each other (2nd and 3rd columns) and slightly different from the measured experimental LogD values (1st column), being the predicted values closer to normal distributions.

Group of compounds In this use case, we selected a few compounds to compare the explanations extracted from two models. Those compounds were filtered from a group of similar structures detailed in Figures 2 and 3.

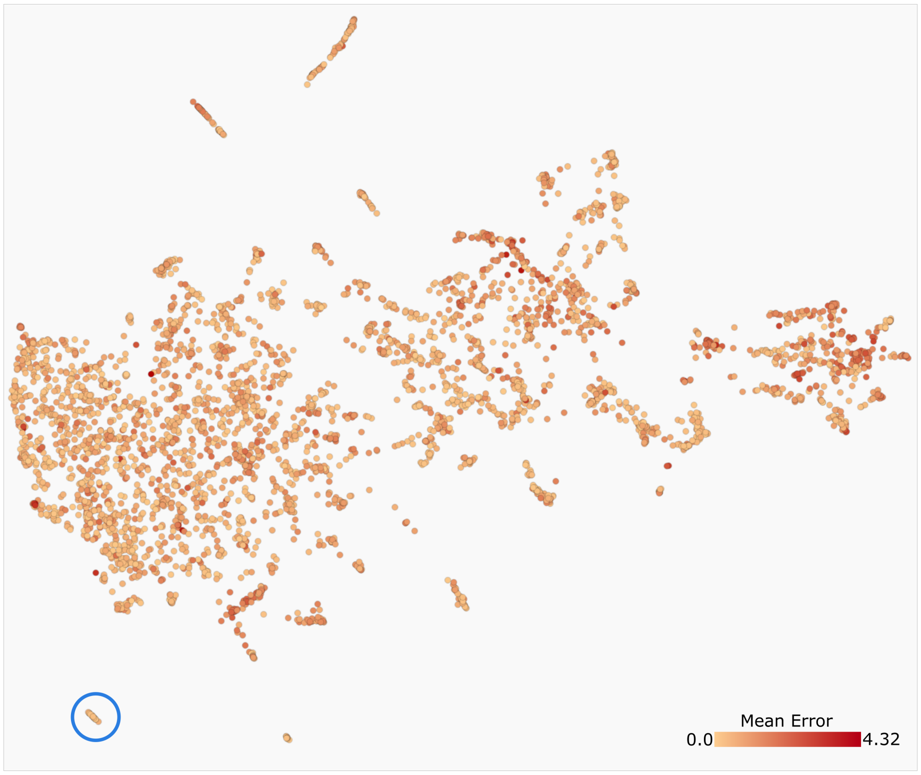

Figure 1: Projection of compounds based on XAI model's latent space representations. Color represents the average between absolute errors from Base and XAI models. The blue circle identifies the studied group; its mean errors appear in Figure 3.

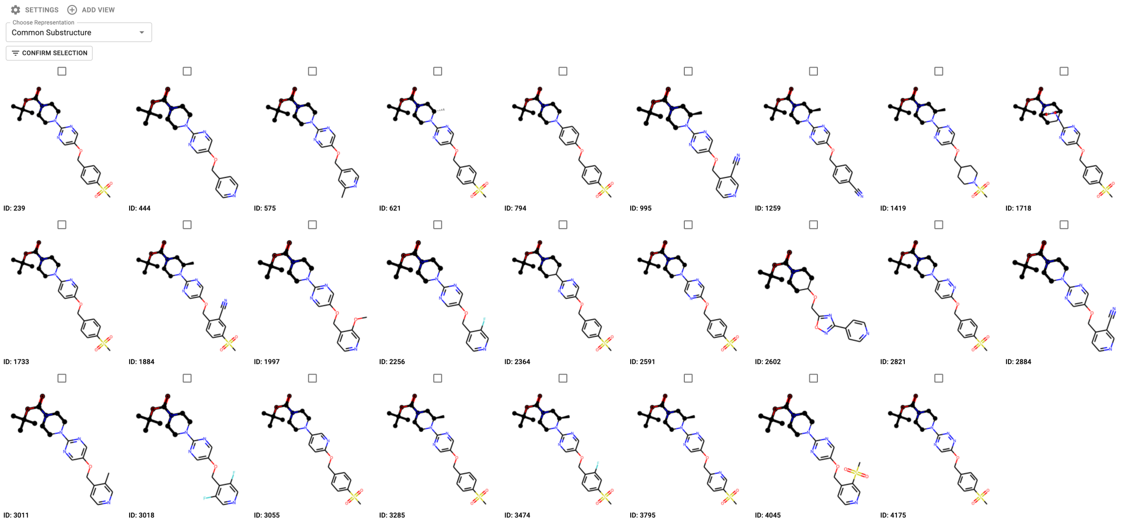

Figure 2: Overview of the compounds in the studied group, as seen in the Summary View in CIME.

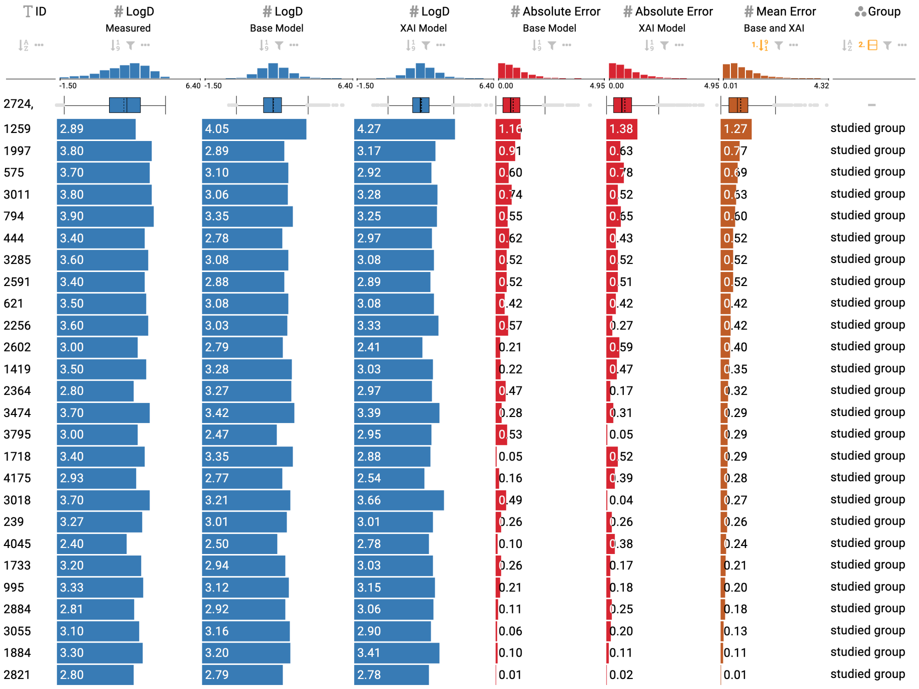

ID	LogD Measured	LogD Base Model	LogD XAI Model	Absolute Error Base Model	Absolute Error XAI Model	Mean Error Base and XAI	Group
1259	2.89	4.05	4.27	1.16	1.38	1.27	studied group
1997	3.80	2.89	3.17	0.91	0.63	0.77	studied group
575	3.70	3.10	2.92	0.60	0.78	0.69	studied group
3011	3.80	3.06	3.28	0.74	0.52	0.63	studied group
794	3.90	3.35	3.25	0.55	0.55	0.60	studied group
444	3.40	2.78	2.97	0.62	0.43	0.52	studied group
3285	3.60	3.08	3.08	0.52	0.52	0.52	studied group
2591	3.40	2.88	2.89	0.52	0.51	0.52	studied group
621	3.50	3.08	3.08	0.42	0.42	0.42	studied group
2256	3.60	3.03	3.33	0.57	0.27	0.42	studied group
2602	3.00	2.79	2.41	0.21	0.59	0.40	studied group
1419	3.50	3.28	3.03	0.22	0.47	0.35	studied group
2364	2.80	3.27	2.97	0.47	0.17	0.32	studied group
3474	3.70	3.42	3.39	0.28	0.31	0.29	studied group
3795	3.00	2.47	2.95	0.53	0.05	0.29	studied group
1718	3.40	3.35	2.88	0.05	0.52	0.29	studied group
4175	2.93	2.77	2.54	0.16	0.39	0.28	studied group
3018	3.70	3.21	3.66	0.49	0.04	0.27	studied group
239	3.27	3.01	3.01	0.26	0.26	0.26	studied group
4045	2.40	2.50	2.78	0.10	0.38	0.24	studied group
1733	3.20	2.94	3.03	0.26	0.17	0.21	studied group
995	3.33	3.12	3.15	0.21	0.18	0.20	studied group
2884	2.81	2.92	3.06	0.11	0.25	0.18	studied group
3055	3.10	3.16	2.90	0.06	0.20	0.13	studied group
1884	3.30	3.20	3.41	0.10	0.11	0.11	studied group
2821	2.80	2.79	2.78	0.01	0.02	0.01	studied group

Figure 3: Detailed screenshot of CIME's "Table View" with ID, measured LogD, predicted LogD (base and XAI models), absolute errors (base and XAI), and mean between errors (base and XAI) of each compound in the studied group (bar charts). Compounds are sorted by mean error. Histograms represent the entire dataset, and box plots represent all compounds that are not in the studied group.

References

[1] Akiba, T., Sano, S., Yanase, T., Ohta, T., Koyama, M.: Optuna: A next-generation hyperparameter optimization framework. In: Proceedings of the 25th ACM SIGKDD International Conference on Knowledge Discovery & Data Mining. KDD '19, pp. 2623–2631. Association for Computing Machinery, New York (2019). doi:10.1145/3292500.3330701. https://doi.org/10.1145/3292500.3330701 Accessed 2021-11-25

[2] Willmott, C.J., Matsuura, K.: Advantages of the mean absolute error (mae) over the root mean square error (rmse) in assessing average model performance. Clim. Res. **30**(1), 79–82 (2005)
